# Botulinum Toxin Type A-assisted Mesh-Free Endoscopic Repair of a Pediatric Ventral Hernia After Giant Omphalocele: A Case Report

**DOI:** 10.7759/cureus.112124

**Published:** 2026-07-06

**Authors:** Juan Diego Vera Proaño, Camila Margarita Díaz Guíñez, Juan Daniel Zurita Estrella, Jacob Raúl Rodríguez Espinoza, Álvaro Morillo Cox, Tatiana Fernandez Trokhimtchouk

**Affiliations:** 1 Pediatric Surgery, Universidad San Francisco de Quito, Quito, ECU; 2 Pediatric Surgery, Hospital Gineco Obstétrico Pediátrico de Nueva Aurora Luz Elena Arizmendi, Quito, ECU; 3 General Surgery, Universidad Internacional del Ecuador, Quito, ECU; 4 General Surgery, Hospital General del Sur de Quito, Instituto Ecuatoriano de Seguridad Social (IESS), Quito, ECU

**Keywords:** abdominal wall reconstruction, botulinum toxin type a, chemical muscle relaxation, endoscopic preaponeurotic repair, giant omphalocele, mesh-free repair, pediatric surgery, ventral hernia

## Abstract

Residual ventral hernia after giant omphalocele presents a complex reconstructive challenge in pediatric surgery because of altered abdominal wall anatomy, muscular retraction, and the need to achieve closure without excessive tension. Botulinum toxin type A has been used as an adjunct to abdominal wall reconstruction by inducing temporary muscular relaxation, but pediatric experience remains limited, particularly in combination with endoscopic preaponeurotic repair (REPA).

We report the case of a five-year-old boy weighing 13.4 kg with a residual ventral hernia after a giant omphalocele. Preoperative ultrasonography demonstrated a 6 cm fascial defect. The patient underwent ultrasound-guided botulinum toxin type A infiltration using 14 injection sites distributed bilaterally along the rectus abdominis and lateral abdominal wall musculature. A total dose of 70 IU was administered. Four weeks later, the defect diameter had decreased to 4 cm, and mesh-free REPA was performed. Complete musculoaponeurotic closure was achieved without prosthetic mesh, progressive preoperative pneumoperitoneum, or surgical component separation.

The postoperative course was uneventful, and no clinical recurrence or wound-related complication was observed at 12 months of follow-up. This case describes a technically successful combination of preoperative botulinum toxin type A infiltration and mesh-free REPA in a child with a post-omphalocele ventral hernia.

## Introduction

Giant omphalocele remains one of the most complex congenital abdominal wall defects in pediatric surgery. In addition to the size of the parietal defect, these patients frequently present with visceral-abdominal disproportion, lateral abdominal wall retraction, and varying degrees of loss of domain, all of which may preclude safe primary closure during the neonatal period [1]. As a result, delayed reconstruction is often required after epithelialization or staged management, and residual ventral hernias may persist into childhood [2].

The reconstruction of post-omphalocele ventral hernias poses a specific technical challenge: the surgeon must restore abdominal wall continuity while avoiding excessive tension, respiratory compromise, and unnecessary morbidity. Traditional options include open repair, prosthetic reinforcement, staged reconstruction, and component separation techniques. Although these strategies can be effective, their use in children raises concerns related to surgical trauma, prosthetic material, future growth, and long-term abdominal wall function [2,3].

Botulinum toxin type A has been increasingly used as an adjunct in complex abdominal wall reconstruction. By inducing temporary relaxation of the abdominal wall musculature, it may improve compliance, reduce fascial tension, and facilitate primary closure. Most evidence comes from adult complex ventral hernia repair, but pediatric experience has gradually emerged, including reports of its use in patients with giant omphalocele or residual post-omphalocele ventral hernias [3,4].

Endoscopic preaponeurotic repair (REPA) offers a minimally invasive approach to midline abdominal wall reconstruction through a subcutaneous/preaponeurotic working space, avoiding entry into the peritoneal cavity and reducing the need for wide open dissection [5]. However, its use in pediatric ventral hernias secondary to giant omphalocele remains scarcely described.

We report the case of a five-year-old boy with a residual ventral hernia after a giant omphalocele who underwent preoperative ultrasound-guided botulinum toxin type A infiltration followed by mesh-free REPA. The main interest of this case is the combination of abdominal wall preparation with botulinum toxin type A and endoscopic preaponeurotic closure in a pediatric post-omphalocele ventral hernia, without progressive preoperative pneumoperitoneum, prosthetic mesh, or surgical component separation.

## Case presentation

A five-year-old boy weighing 13.4 kg was evaluated for a residual ventral hernia after previous treatment of a giant omphalocele. On physical examination, a midline abdominal wall defect was evident, with protrusion of the hernia sac and retraction of the surrounding abdominal wall musculature. Preoperative ultrasonography demonstrated a fascial defect measuring 6 cm in transverse diameter.

**Figure 1:**
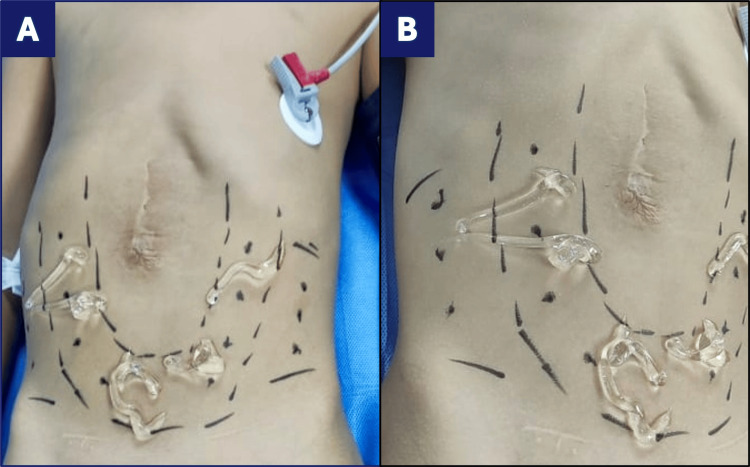
Preoperative marking of botulinum toxin type A injection sites. (A) Frontal view showing the residual midline ventral hernia after a giant omphalocele and bilateral abdominal wall markings. (B) Oblique view showing the distribution of the injection sites along the rectus abdominis and lateral abdominal wall musculature. Four injection points were marked along each rectus abdominis muscle and three additional points along the lateral abdominal wall musculature bilaterally, for a total of 14 injection sites.

**Figure 2:**
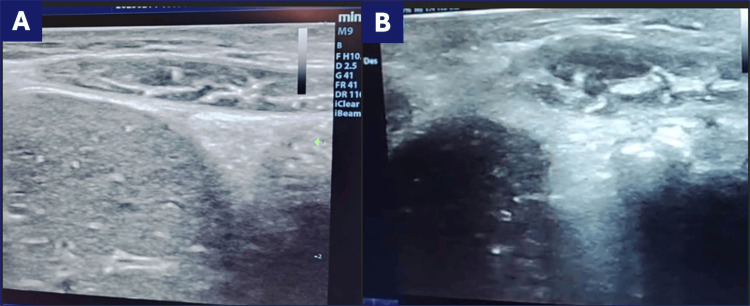
Ultrasound-guided botulinum toxin type A infiltration. (A) Identification of the selected abdominal wall muscle plane under ultrasound guidance. (B) Intramuscular injection of botulinum toxin type A, with local separation of the muscle fibers confirming delivery within the intended plane.

Botulinum toxin type A (Otesaly) was administered under general anesthesia. Four injection points were marked along each rectus abdominis muscle and three additional points along the lateral abdominal wall musculature on each side, for a total of 14 injection sites (Figure 1). Under ultrasound guidance, 5 IU were infiltrated at each site, for a total dose of 70 IU, corresponding to 5.2 IU/kg. Ultrasound guidance was used to confirm the muscular plane during injection (Figure 2).

**Figure 3:**
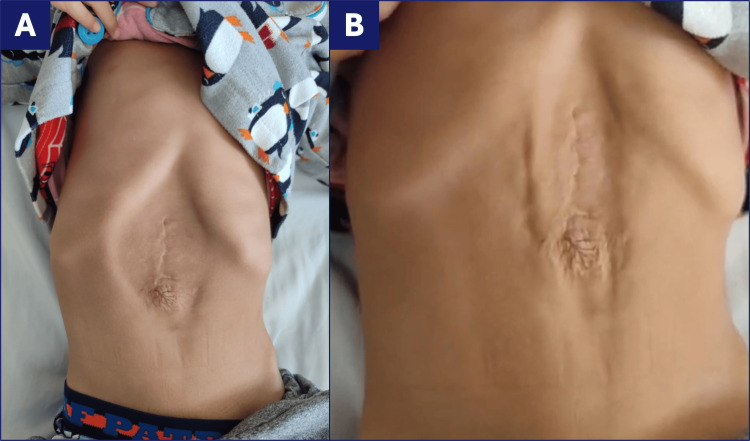
Clinical appearance before and after botulinum toxin type A infiltration. (A) Pre-infiltration appearance showing the residual midline ventral hernia after a giant omphalocele. (B) Clinical appearance four weeks after botulinum toxin type A infiltration, with improved abdominal wall contour before definitive endoscopic repair.

Four weeks after infiltration, the patient was reassessed clinically and by ultrasonography. The abdominal wall appeared more compliant, with reduced muscular retraction, and the transverse diameter of the fascial defect decreased from 6 cm to 4 cm (Figure 3). Based on these findings, definitive REPA was performed.

**Figure 4:**
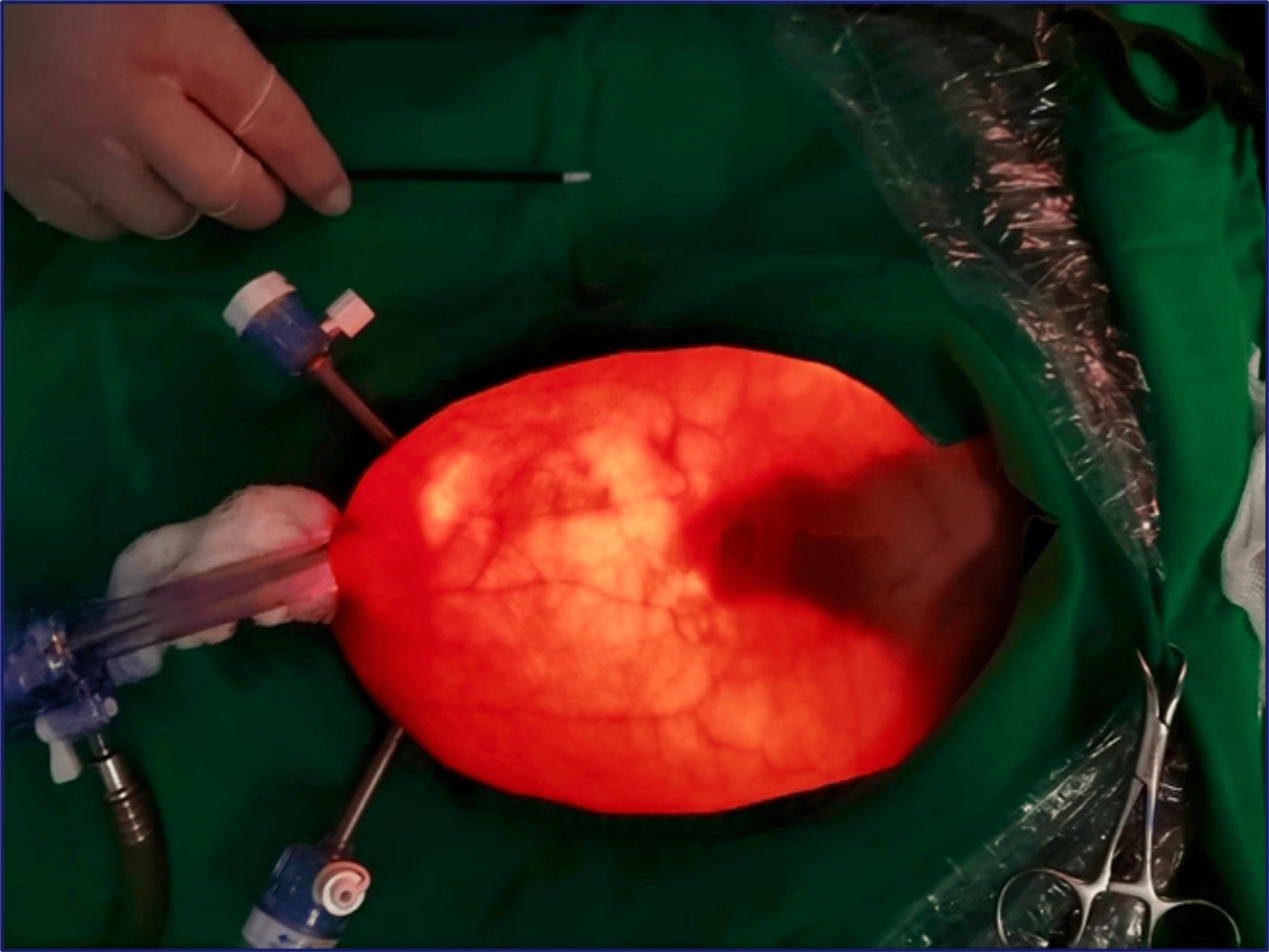
Trocar placement for endoscopic preaponeurotic repair. A 12-mm suprapubic trocar was used for optical access, and two 5-mm suprapubic working ports were placed bilaterally to create and dissect the preaponeurotic working space.

**Figure 5:**
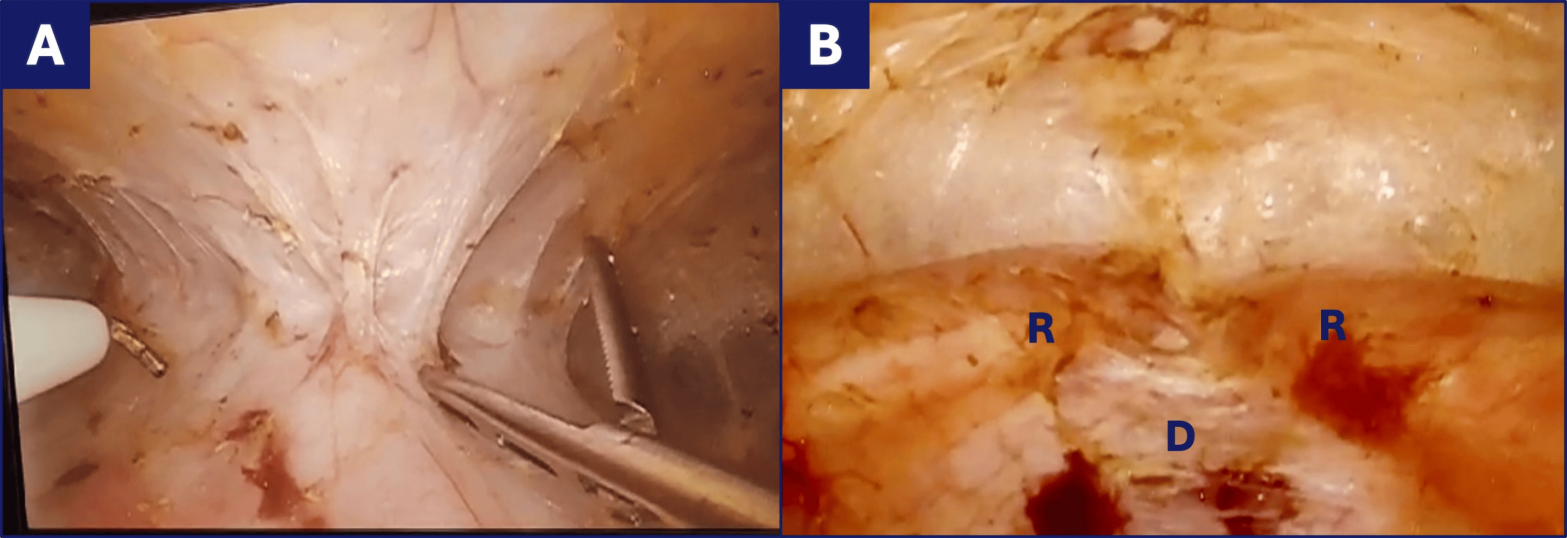
Endoscopic preaponeurotic dissection and exposure of the abdominal wall defect. (A) Development of the preaponeurotic plane over the anterior abdominal wall. (B) Exposure of the residual ventral hernia defect after dissection. R: rectus abdominis muscle; D: abdominal wall defect.

A suprapubic 12-mm trocar and two bilateral 5-mm working ports were placed to access the preaponeurotic plane (Figure 4). Carbon dioxide insufflation was used to create the working space, and the subcutaneous tissue was dissected from the anterior aponeurosis from the xiphoid process to the pubis, preserving vascular structures and the hernia sac. The dissection allowed complete exposure of the musculoaponeurotic margins of the defect (Figure 5).

**Figure 6:**
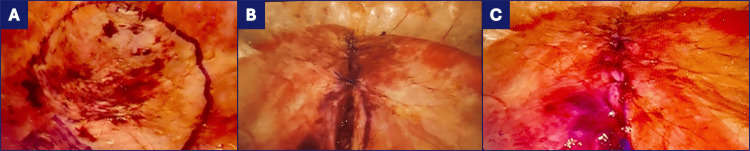
Endoscopic closure of the abdominal wall defect. (A) Delineation of the residual musculoaponeurotic defect after preaponeurotic dissection. (B) Initial approximation of the defect with interrupted nonabsorbable monofilament sutures. (C) Final endoscopic appearance after continuous closure with barbed 3-0 polydioxanone suture.

The areas of greatest tension were initially approximated with interrupted 2-0 polypropylene sutures. Definitive closure was then completed with a continuous barbed 3-0 polydioxanone suture, achieving complete approximation of the abdominal wall defect under endoscopic vision (Figure 6). No prosthetic mesh was implanted, and no surgical component separation was required. A tubular drain was left in the preaponeurotic space.

The postoperative course was uneventful. Drain output was scarce and serous, and the drain was removed on postoperative day 7. The patient was discharged after clinical evaluation showed adequate recovery and no evidence of early recurrence. At 12 months of follow-up, the patient remained asymptomatic, with satisfactory cosmetic evolution and no clinical evidence of recurrence or wound-related complications.

## Discussion

Residual ventral hernia after giant omphalocele is a complex reconstructive problem because the defect is usually associated with previous visceral-abdominal disproportion, altered abdominal wall anatomy, scarred midline tissue, and retraction of the muscular components. Delayed reconstruction is frequently required when primary neonatal closure is not feasible, and several strategies have been described, including staged closure, prosthetic reinforcement, tissue expansion, component separation, progressive pneumoperitoneum, and, more recently, preoperative botulinum toxin type A infiltration [2,6,7].

Current pediatric experience with botulinum toxin type A in this context remains limited and heterogeneous. Published reports include its use as part of proposed protocols for post-omphalocele ventral hernia repair, in combination with progressive preoperative pneumoperitoneum, as an adjunct in neonatal giant omphalocele repair, and in selected complex pediatric ventral hernias [2,3,6,8,9]. These reports support the feasibility of botulinum toxin type A as an adjunct to abdominal wall reconstruction in selected pediatric patients, but they do not establish a standardized protocol regarding dose, injection distribution, timing, or associated reconstructive technique.

The first relevant decision in this case was to use botulinum toxin type A before definitive repair. The rationale was to improve abdominal wall compliance and reduce closure tension before attempting primary musculoaponeurotic approximation. This concept has been described in adult complex ventral hernia surgery as “chemical component relaxation,” with systematic reviews reporting improved fascial medialization and facilitation of primary closure when botulinum toxin type A is used before abdominal wall reconstruction [4,7]. Although most evidence comes from adult populations, pediatric reports suggest that this strategy can be considered in selected children with giant omphalocele or residual post-omphalocele ventral hernias [2,3,6,8,9].

In the present patient, botulinum toxin type A was administered four weeks before definitive repair. This interval was chosen because most published protocols perform reconstruction approximately three to six weeks after infiltration, allowing time for the toxin-induced muscular relaxation to develop before surgery [2,4]. After infiltration, the transverse diameter of the defect decreased from 6 cm to 4 cm, and the abdominal wall appeared clinically more compliant. These findings supported proceeding with definitive closure without additional preoperative expansion maneuvers.

The injection protocol deserves specific consideration. Most published descriptions of chemical component relaxation target the lateral abdominal wall muscles, particularly the external oblique, internal oblique, and transversus abdominis muscles [4,7]. In this case, the authors used 14 injection sites distributed bilaterally, with four points along each rectus abdominis muscle and three points along the lateral abdominal wall musculature on each side. This pattern differs from the most commonly described protocols. Therefore, the present case should not be interpreted as evidence that this is the optimal injection map, but rather as a technically successful use of a low-dose, ultrasound-guided protocol in one pediatric patient.

The second decision was to avoid progressive preoperative pneumoperitoneum. Rombaldi et al. reported the closest comparable pediatric case: a seven-year-old child with a large post-omphalocele ventral hernia measuring approximately 9 cm and containing liver, treated with botulinum toxin type A followed by progressive preoperative pneumoperitoneum and open repair without mesh or component separation [3]. In contrast, the present patient had a smaller defect and demonstrated a reduction in defect diameter after botulinum toxin infiltration. Definitive closure was therefore attempted without progressive pneumoperitoneum, avoiding an additional invasive step.

The third decision was to perform the repair through an endoscopic preaponeurotic approach. REPA was originally described as a minimally invasive technique for rectus diastasis and selected midline ventral hernias, using a preaponeurotic working space to allow anterior midline plication and reinforcement while avoiding a large open incision [5]. In this case, the same anatomical plane was used to expose the residual post-omphalocele defect and approximate the musculoaponeurotic margins under endoscopic vision. This approach was selected because the defect was anterior, midline, and accessible from the preaponeurotic space.

The fourth decision was to avoid prosthetic mesh. Although mesh reinforcement is widely used in adult ventral hernia repair and in many REPA procedures, its role in pediatric abdominal wall reconstruction remains more selective. In children, the use of prosthetic material must be balanced against the possibility of infection, foreign-body complications, and uncertainty regarding long-term behavior during growth [2]. In this case, botulinum toxin type A infiltration reduced the defect diameter and improved tissue approximation sufficiently to permit mesh-free closure.

The fifth decision was to avoid surgical component separation. Component separation has been reported as a reconstructive option in patients with giant omphalocele and large residual abdominal wall defects, particularly when primary midline approximation cannot otherwise be achieved [10]. However, it requires additional dissection and release of abdominal wall components. In the present patient, the combination of preoperative botulinum toxin type A and endoscopic preaponeurotic access allowed complete closure without formal component separation.

The main interest of this report is the combination of preoperative botulinum toxin type A infiltration with mesh-free REPA in a child with residual ventral hernia after giant omphalocele. To our knowledge, pediatric reports have described botulinum toxin type A in this setting, including in combination with progressive preoperative pneumoperitoneum or other reconstructive strategies, but not specifically with REPA [2,3,6,8,9]. Therefore, this case adds a technical variant to the limited pediatric literature on abdominal wall preparation before delayed reconstruction after giant omphalocele.

The limitations of this report are inherent to its single-case design. The findings cannot establish safety, efficacy, reproducibility, or superiority over other reconstructive strategies. In addition, objective imaging data were limited to transverse defect diameter, the injection distribution was not standardized according to previously published protocols, and long-term functional abdominal wall outcomes were not formally assessed. Although the 12-month clinical follow-up was favorable, longer observation is needed to evaluate durability, recurrence, growth-related changes, cosmesis, and abdominal wall function.

This case documents a technically successful sequence of management in one child: preoperative ultrasound-guided botulinum toxin type A infiltration, followed by mesh-free endoscopic preaponeurotic closure without progressive preoperative pneumoperitoneum or surgical component separation. Further pediatric experience is required to determine whether this approach is reproducible, which patients may benefit, and what dosing, injection mapping, timing, imaging assessment, and follow-up parameters should be used.

## Conclusions

This case describes the use of preoperative ultrasound-guided botulinum toxin type A infiltration followed by mesh-free REPA in a five-year-old boy with residual ventral hernia after giant omphalocele. In this patient, the sequence was followed by a reduction in defect diameter, complete endoscopic musculoaponeurotic closure, and no clinical recurrence at 12 months of follow-up. The main relevance of this report is the combination of botulinum toxin type A with an endoscopic preaponeurotic approach in a pediatric post-omphalocele ventral hernia, without progressive preoperative pneumoperitoneum, prosthetic mesh, or surgical component separation. This case adds a technical alternative to the limited pediatric experience with abdominal wall preparation before delayed reconstruction after a giant omphalocele.

Further pediatric cases with standardized imaging measurements, dosing protocols, injection mapping, and longer follow-up are needed to better define the role of this approach in abdominal wall reconstruction.
